# Right Ventricular Function Predicts Adverse Clinical Outcomes in Patients With Chronic Thromboembolic Pulmonary Hypertension: A Three-Dimensional Echocardiographic Study

**DOI:** 10.3389/fmed.2021.697396

**Published:** 2021-08-23

**Authors:** Yidan Li, Lirong Liang, Dichen Guo, Yuanhua Yang, Juanni Gong, Xinyuan Zhang, Di Zhang, Zhe Jiang, Xiuzhang Lu

**Affiliations:** ^1^Department of Echocardiography, Heart Center, Beijing Chao-Yang Hospital, Capital Medical University, Beijing, China; ^2^Clinical Epidemiology & Tobacco Dependence Treatment Research Department, Beijing Institute of Respiratory Medicine, Beijing Chao-Yang Hospital, Capital Medical University, Beijing, China; ^3^Department of Respiratory and Critical Care Medicine, Beijing Chao-Yang Hospital, Capital Medical University, Beijing, China

**Keywords:** three-dimensional echocardiography, machine learning, right ventricular function, pulmonary hypertension, clinical failure, prognosis

## Abstract

**Background:** Right ventricular (RV) function plays a vital role in the prognosis of patients with chronic thromboembolic pulmonary hypertension (CTEPH). We used new machine learning (ML)-based fully automated software to quantify RV function using three-dimensional echocardiography (3DE) to predict adverse clinical outcomes in CTEPH patients.

**Methods:** A total of 151 consecutive CTEPH patients were registered in this prospective study between April 2015 and July 2019. New ML-based methods were used for data management, and quantitative analysis of RV volume and ejection fraction (RVEF) was performed offline. RV structural and functional parameters were recorded using 3DE. CTEPH was diagnosed using right heart catheterization, and 62 patients underwent cardiac magnetic resonance to assess right heart function. Adverse clinical outcomes were defined as PH-related hospitalization with hemoptysis or increased RV failure, including conditions requiring balloon pulmonary angioplasty or pulmonary endarterectomy, as well as death.

**Results:** The median follow-up time was 19.7 months (interquartile range, 0.5–54 months). Among the 151 CTEPH patients, 72 experienced adverse clinical outcomes. Multivariate Cox proportional-hazard analysis showed that ML-based 3DE analysis of RVEF was a predictor of adverse clinical outcomes (hazard ratio, 1.576; 95% confidence interval (CI), 1.046~2.372; *P* = 0.030).

**Conclusions:** The new ML-based 3DE algorithm is a promising technique for rapid 3D quantification of RV function in CTEPH patients.

## Introduction

Chronic thromboembolic pulmonary hypertension (CTEPH) is a subgroup in the classification of PH, which differs from other types of PH with regards to patient characteristics, pathophysiology, and treatment (1). CTEPH is a rare pulmonary vascular disease and usually results from acute pulmonary embolism. CTEPH can cause progressive PH and increase right heart pressure. If not treated appropriately, CTEPH patients will quickly develop right heart failure or even death (2). The overall incidence of CTEPH is 8~37.8 cases per million patients, and the probability of developing CTEPH after acute pulmonary embolism is 0.5~5.1% (3–5). A number of factors, including mechanical obstruction and secondary pulmonary vascular disease, may cause PH and right ventricular (RV) remodeling and dilation, eventually leading to dyspnea, deterioration of exercise tolerance, syncope, and fatigue.

CTEPH disease progression is accompanied by different degrees of myocardial hypertrophy, and right heart failure is the primary cause of death in CTEPH patients. RV fibrosis is a sign of potential maladaptation as a result of increased afterload caused by myocardial PH (6). Cardiac hypertrophy, myocardial fibroblast apoptosis, and RV remodeling result in decreased myocardial contractile function and impaired cardiac pump function, eventually leading to right heart failure.

The right cardiac chambers and ancillary structures have long been neglected in research, but an increasing number of studies have shown that the structure and function of the right heart predicts the prognosis of CTEPH patients. Three-dimensional echocardiography (3DE) allows accurate and repeatable measurements of RV size and function. Most recently, the application of artificial intelligence methods, including machine learning (ML) algorithm techniques, have been developed to automatically detect RV intima boundaries (7–9). The ML-based 3DE algorithm provides fast image editing, thus allowing for the measurement of cardiac cavity volume with good repeatability, offering a promising solution for fast 3D quantification of RV function (10, 11). The objective of this study was to evaluate ML-based 3DE quantification of RV function as a predictor of adverse clinical outcomes in CTEPH patients.

## Materials and Methods

### Ethics Statement

This prospective study was conducted according to the principles defined in the Declaration of Helsinki. The study protocol was approved by the Ethics Committee at Beijing Chao-yang Hospital. All participants in this study signed written informed consent forms prior to the initiation of this study.

### Patient Selection and Study Protocol

The diagnostic criteria of CTEPH were as follows: at rest, the mean pulmonary arterial pressure measured by the right heart catheter was ≥25 mmHg and pulmonary wedge pressure (PAWP) was ≤15 mmHg; ventilation/perfusion (V/Q) imaging had at least one large perfusion defect at one segment or two subsections; or the presence of pulmonary vascular lesions identified by computed tomography (CT), magnetic resonance imaging (MRI), and/or pulmonary angiography (12).

Clinical evaluation, echocardiography, 6-minute walk test (6MWT), and laboratory biochemical tests at 48 h intervals (N-terminal pro-B-type natriuretic peptide, NT-proBNP) were collected and recorded from all participants. Sixty-two patients underwent cardiac magnetic resonance (CMR) examination within 72 h to determine RV volume and ejection fraction (RVEF).

**Figure 1:**
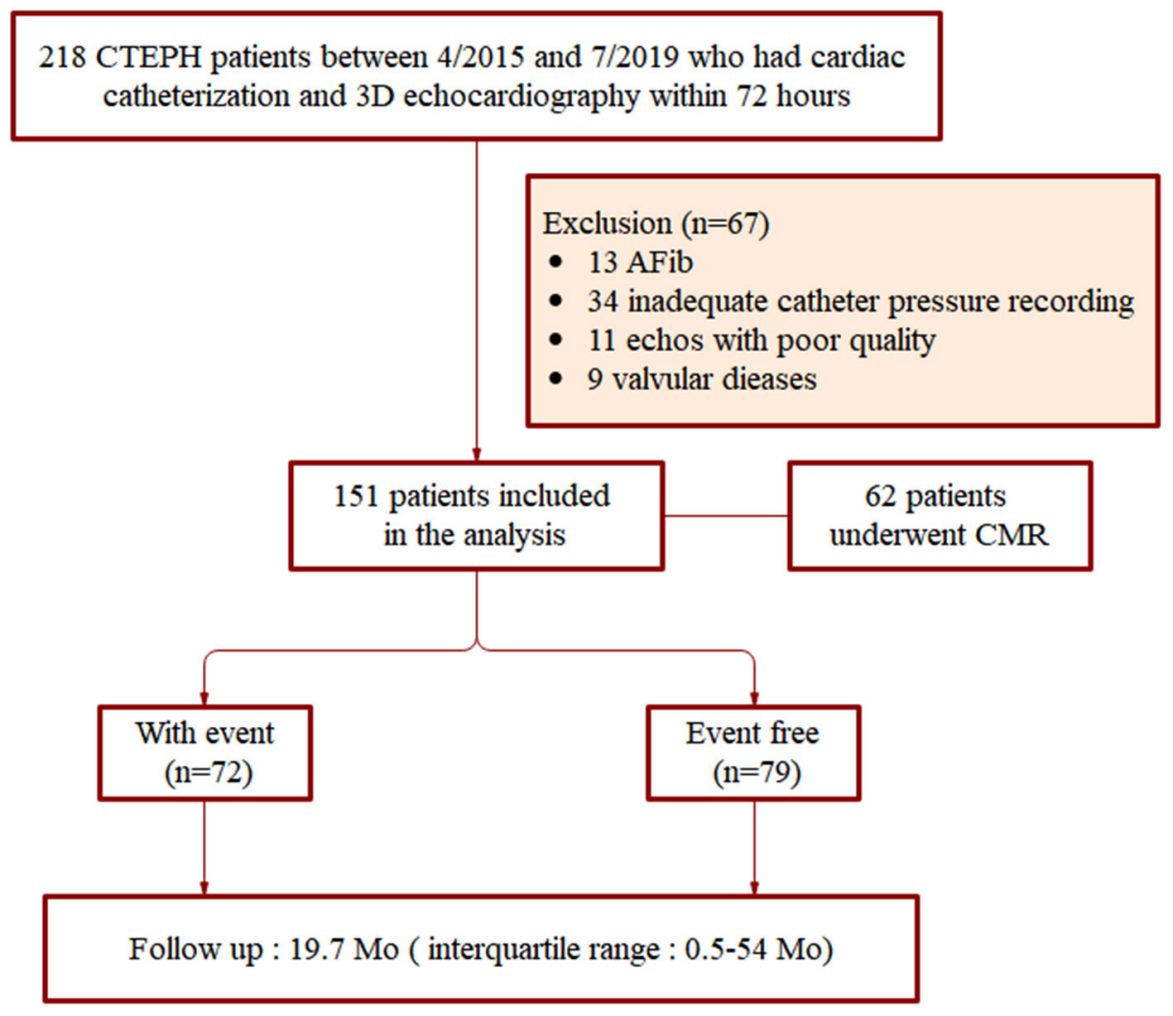
Flow chart showing patient selection process.

Demographic and baseline clinical characteristics of patients were obtained through hospital databases and telephone follow-up. Exclusion criteria included: incomplete clinical and catheter pressure data, atrial fibrillation, moderate or severe aortic and/or mitral valvular heart disease, and poor quality of echocardiographic images. The study began with the inclusion of 218 CTEPH patients. Among the original cohort, 34 patients did not have complete clinical and catheter pressure recording, 13 had atrial fibrillation, nine had valvular heart disease. There were 11 patients (5.04%) who failed to obtain satisfactory 3D parameters due to poor image quality that could not clearly track the endocardium. After application of the exclusion criteria, 151 patients were finally included in this study between April 2015 and July 2019 (Figure 1).

### 3DE Imaging and Analysis

Echocardiographic imaging was performed with a Philips EPIQ 7C (Philips Healthcare, MA, USA). An X5-1 transducer (1–5 MHz) was equipped onto a Doppler ultrasound machine. In the breath-holding state, 3DE images were collected over a maximum of 6 beats in an apical four-chamber (4Ch) RV-focused dataset. Throughout the cardiac cycle scanning, the entire RV cavity was within the scan volume. The imaging depth and sector width were optimized for the maximum frame rate and the 3DE dataset was analyzed offline.

**Figure 2:**
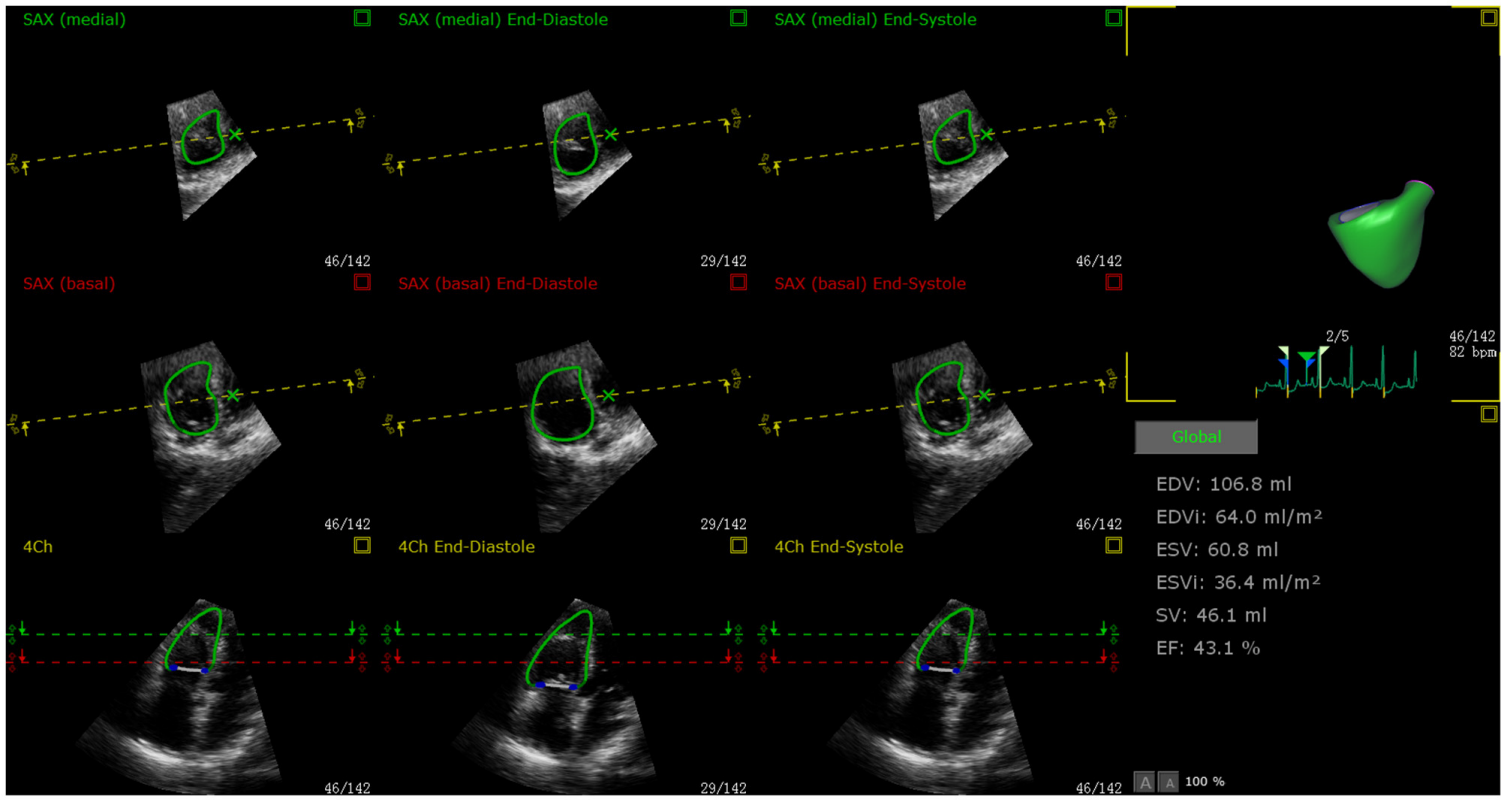
Example of ML-based 3D RVEF analysis using QLAB 13.0 software. RVEF calculation in a 48-year-old female CTEPH patient who was treated with riociguat. End-diastolic and systolic verification and editing of endocardial RV borders, from which RVEDV, RVESV, and RVEF were obtained.

Quantitative analysis of RV function was performed using a new ML method (3D Auto RV, Philips Healthcare) for offline data management (QLAB, Philips). According to the operating instructions of the software, a 3D dynamic surface model of the RV was generated, from which the RV end-diastolic volume (RVEDV) and end-systolic volume (RVESV) were fully-automated. RVEF was calculated according to the formula RVEF = (RVEDV–RVESV)/RVEDV (Figure 2).

### RV Size and Function

The following RV structural parameters measured using 3DE were recorded: RVEDV, RVEDV/body surface area (BSA), RVESV, RVESV/BSA, RV basal diameter, RV mid-diameter, RV longitudinal diameter, RV basal diameter/LV basal diameter, and LV end-diastolic eccentricity index. Pericardial effusion was also recorded.

RV function was evaluated according to the 2015 American Society of Echocardiography guidelines for cardiac chamber quantification by echocardiography in adults (13), including RV index of myocardial performance, RV longitudinal strain (Global & Freewall), tricuspid annular plane systolic excursion (TAPSE), RV fractional area change, and Doppler-derived tricuspid lateral annular systolic velocity (S′).

### CMR Image Analysis

CMR was performed with a 3.0 Tesla magnetic resonance scanner (Magnetom Prisma 3.0-T; Siemens, Erlangen, Germany) using a four-channel cardiac phased-array surface coil for data acquisition. All CMR data were transferred to a workstation (Syngo via, VA30; Siemens, Berlin, Germany) and analyzed with validated software (Cardiac analysis; Siemens Medical Systems, Erlangen, Germany). CMR was performed with the true fast imaging with the steady-state precession sequence (True FISP; repetition time 3.0–3.2 ms, echo time 1.4 ms; flip angle, 70°; field of view 320 × 360 mm, matrix size 256 × 256 mm, phases per cardiac cycle 25; section thickness, 6 mm) using retrospective electrocardiogram triggered during breath holding. The acquisition time per patient was 6–10 min. An experienced radiologist, who identified the endocardial and epicardial boundaries, semi-automatically segmented the right and left ventricles. The RV cavity included endocardial trabeculae. Disk summation was used to calculate RVEDV and RVESV, and RVEF was calculated using the standard formula mentioned above.

### Right Heart Catheterization Procedure

A 7.5-French gauge Swan-Ganz thermodilution catheter (Edwards Lifesciences, Irvine, CA, USA) was inserted into the right inferior pulmonary artery through the internal jugular vein. The following parameters were measured under stable hemodynamics: RA pressure, mean PA pressure, pulmonary vascular resistance, and cardiac index. Cardiac output (CO) undergoing cardiac catheterization was measured using the thermodilution method. PVR in Wood units (WU) was calculated using the equation: PVR = (mean pulmonary arterial pressure (mPAP)–pulmonary artery wedge pressure)/CO. Echocardiography and RHC were performed with an interval of <72 h for all the patients.

### Adverse Clinical Outcomes

The primary end points in this study included the occurrence of adverse clinical outcomes defined as PH-related hospitalization with hemoptysis or increased RV failure, including conditions requiring balloon pulmonary angioplasty or pulmonary endarterectomy, as well as death. The cause of hospitalization or death was decided after reviewing the relevant medical history and documentation. Patients were all followed up until the occurrence of adverse clinical outcomes or the end of the study period.

### Statistical Analysis

All statistical analyses were performed using SPSS Version 23 (SPSS Software, Chicago, IL), R version 3.6.3 (R Foundation for Statistical Computing, Vienna, Austria), and MedCalc 16.1 (MedCalc Software, Mariakerke, Belgium). The one-sample Kolmogorov-Smirnov test was used to verify the normal distribution of all data. Abnormal data are expressed as frequency and percentage or mean ± standard deviation or median (interquartile range). The independent sample *t*-test was used to determine the significant difference of normally distributed data between groups. Pearson or Spearman correlation coefficients were used to assess the relationship between RVEF and other clinical and echocardiographic variables.

Univariate Cox proportional hazards analysis was used to examine the association between the variables and the combined clinical endpoints, with results expressed as hazard ratios and bilateral 95% confidence intervals (CIs). Multivariate survival analysis included all variables with a *P*-value < 0.10 in univariate analysis and prognostic parameters described previously. The survival receiver operating characteristic (ROC) curve was constructed to assess the sensitivity and specificity of predictors used to predict adverse clinical outcomes. The optimal survival threshold was predicted using the Youden's index method. Kaplan-Meier analysis was used to divide patients into quartiles based on RVEF. The incremental value of RVEF over baseline clinical models was estimated by the change in C statistic, whereas, the −2 log-likelihood test was used to estimate the relative fit of each model. We analyzed the consistency between CMR and 3DE measurements of RVEDV and RVESV. A bilateral *P*-value < 0.05 was considered statistically significant.

## Results

### Demographic and Baseline Clinical Characteristics of Participants

A total of 151 CTEPH patients (71.5% females; mean age, 50.9 ± 14.1 years) and 30 age- and sex-matched healthy control subjects (70% females; mean age, 48.3 ± 9.4 years) constituted the study population (Table 1). All patients and control subjects underwent echocardiography, and 62 patients also underwent CMR. A large proportion of patients had cardiac function based on the World Health Organization (WHO) functional class II and III. All patients received anticoagulant therapy (rivaroxaban/warfarin), and 97 patients were on riociguat, 38 were on phosphodiesterase-5 (PDE-5) inhibitors, 31 were on endothelin-1 inhibitors, and 19 were on a combined therapy.

**Table 1:** Demographic and baseline clinical characteristics in CTEPH patients with and without adverse clinical outcomes.

**Variable**	**Study cohort** **(** ***N*** **= 151)**	**Adverse clinical events**		***P*** **-value**
		**Event free** **(** ***n*** **= 79)**	**With event** **(** ***n*** **= 72)**	
Age (y)	50.9 ± 14.1	51.6 ± 13.9	50.2 ± 14.3	0.550
Male, *n* (%)	43 (28.5)	21 (26.5)	22 (30.5)	0.589
Body surface area (m^2^)	1.7 ± 0.2	1.7 ± 0.2	1.7 ± 0.2	0.905
WHO functional class				**0.003**
I	6 (4.0)	6 (7.6)	0 (0)	
II	68 (45.0)	46 (58.2)	22 (30.6)	
III	60 (39.7)	27 (34.2)	33 (45.8)	
IV	17 (11.3)	0 (0)	17 (23.6)	
Disease duration (mo)	21.8 (2–168)	11.5 (2–34)	33.0 (3–168)	**<0.0001**
6-min walk distance (m)	372 (98–630)	412 (196–630)	328 (190–410)	**<0.0001**
NT-proBNP (pg/ml)	941.54 ± 1075.7	646.9 ± 1039.3	1971.1 ± 1755.6	**<0.0001**
Basic hemodynamics data				
RA pressure (mm Hg)	5.9 ± 4.6	4.6 ± 3.6	7.4 ± 5.3	**<0.0001**
Mean PA pressure (mm Hg)	47.4 ± 12.5	44.2 ± 12.3	51.0 ± 11.7	**0.001**
Cardiac index (L/min per m^2^)	2.5 ± 0.8	2.8 ± 0.9	2.2 ± 0.6	**<0.0001**
Pulmonary vascular resistance (wood units)	10.3 ± 5.2	8.1 ± 12.9	24.6 ± 21.9	0.278
PH medical treatment				0.203
Rivaroxaban/Warfarin	151 (100)	79 (100)	72 (100)	
Riociguat	97 (64.2)	51 (64.6)	46 (63.9)	
PDE-5 inhibitors	38 (25.2)	18 (22.8)	20 (27.8)	
Endothelin-1 inhibitors	31 (20.5)	14 (17.7)	17 (23.6)	
Combined therapy	19 (12.6)	6 (7.6)	13 (18.1)	
Follow-up (mo)	19.7 (0.5–54)	27.9 (1–54)	10.7 (0.5–25)	**<0.0001**

*CTEPH, chronic thromboembolic pulmonary hypertension; NT-proBNP, N-terminal pro–B-natriuretic peptide; RA, right atrium; PA, pulmonary artery; PAH, pulmonary arterial hypertension; PDE-5, phosphodiesterase-5. Data are expressed as mean ± SD, number (percentage), or median (interquartile range). P-values indicate significance between patients with and those without adverse clinical outcomes. Bold values indicate statistical significance of the tested parameters*.

### RV Function Measured by ML-Based 3DE

**Figure 3:**
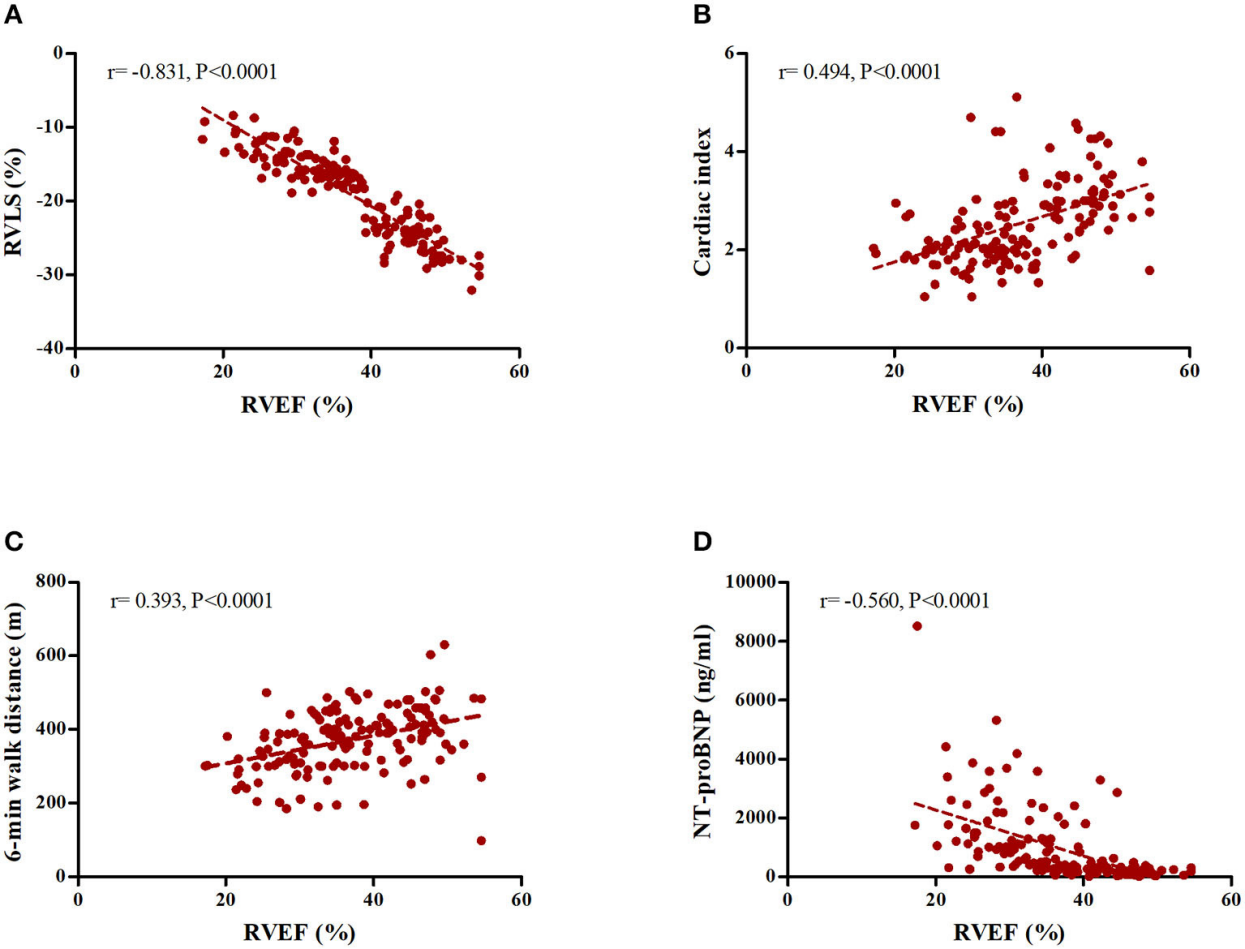
Correlation between ML-based 3D RVEF and physiological parameters. **(A)** RV longitudinal strain (Free wall). **(B)** Cardiac index. **(C)** 6MWD. **(D)** NT-proBNP.

The echocardiographic features of participants measured by ML-based 3DE are summarized in Table 2. CTEPH patients had significantly lower RVEF values compared to the control subjects (36.8 ± 8.6 vs. 52.4 ± 3.2%, *P* < 0.0001). We found a correlation between RVEF and basic function parameters. For example, RVEF was positively associated with cardiac index (*r* = 0.494, *P* < 0.0001) and 6MWD (*r* = 0.393, *P* < 0.0001), but negatively associated with NT-proBNP (*r* = −0.560, *P* < 0.0001). In addition, there was a strong negative correlation between RVEF and RV longitudinal strain (free wall) (*r* = −0.831, *P* < 0.0001) (Figure 3).

**Table 2:** RV indices in CTEPH patients with and without adverse clinical outcomes.

**Variable**	**Study cohor**t **(*****N*****= 151)**	**Adverse clinical outcomes**		***P*** **-value**
		**Event free** **(** ***n*** **= 79)**	**With event** **(** ***n*** **= 72)**	
RV functional parameters				
RVEF (%)	36.8 ± 8.6	42.3 ± 6.3	30.9 ± 6.9	**<0.0001**
RV longitudinal strain (global) (%)	−14.9 ± 4.2	−16.8 ± 3.5	−12.7 ± 3.9	**<0.0001**
RV longitudinal strain (Free wall) (%)	−18.6 ± 6.2	−22.1 ± 4.8	−14.8 ± 5.3	**<0.0001**
TAPSE (mm)	15.6 ± 5.4	18.1 ± 4.9	13.0 ± 4.6	**<0.0001**
RV fractional area change (%)	27.9 ± 9.1	32.6 ± 8.0	22.8 ± 7.3	**<0.0001**
RV index of myocardial performance	0.7 ± 0.2	0.6 ± 0.2	0.8 ± 0.2	**<0.0001**
S' (cm/s)	10.7 ± 3.1	11.8 ± 3.1	9.5 ± 2.6	**<0.0001**
RV structural parameters				
RV end-diastolic volume (ml)	123.1 ± 34.8	113.7 ± 28.2	133.4 ± 38.4	**0.001**
RV end-diastolic volume indexed to BSA (ml/m^2^)	72.2 ± 19.3	66.8 ± 16.0	78.1 ± 20.9	**<0.0001**
RV end-systolic volume (ml)	79.0 ± 29.6	66.0 ± 18.9	93.3 ± 32.6	0.201
RV end-systolic volume indexed to BSA (ml/m^2^)	46.3 ± 16.8	38.9 ± 11.3	54.6 ± 18.0	**<0.0001**
RV basal diameter (mm)	45.7 ± 6.7	43.8 ± 6.3	47.8 ± 6.6	**<0.0001**
RV mid-diameter (mm)	42.4 ± 7.9	40.0 ± 5.9	45.1 ± 8.9	**<0.0001**
RV longitudinal diameter (mm)	74.8 ± 8.2	74.3 ± 7.3	75.5 ± 9.1	0.359
RV basal diameter/LV basal diameter	1.3 ± 0.3	1.2 ± 0.3	1.4 ± 0.3	**<0.0001**
LV end-diastolic eccentricity index	1.4 ± 0.3	1.3 ± 0.2	1.5 ± 0.3	**<0.0001**
Pericardial effusion	77 (51.0)	29 (36.7)	48 (66.7)	**0.002**

*RVEF, right ventricular ejection fraction; TAPSE, tricuspid annular plane systolic excursion; BSA, body surface area; LV, left ventricular. Data are expressed as mean ± SD. P-values indicate statistical significance between patients with and without adverse clinical outcomes. Bold values indicate statistical significance of the tested parameters*.

**Figure 4:**
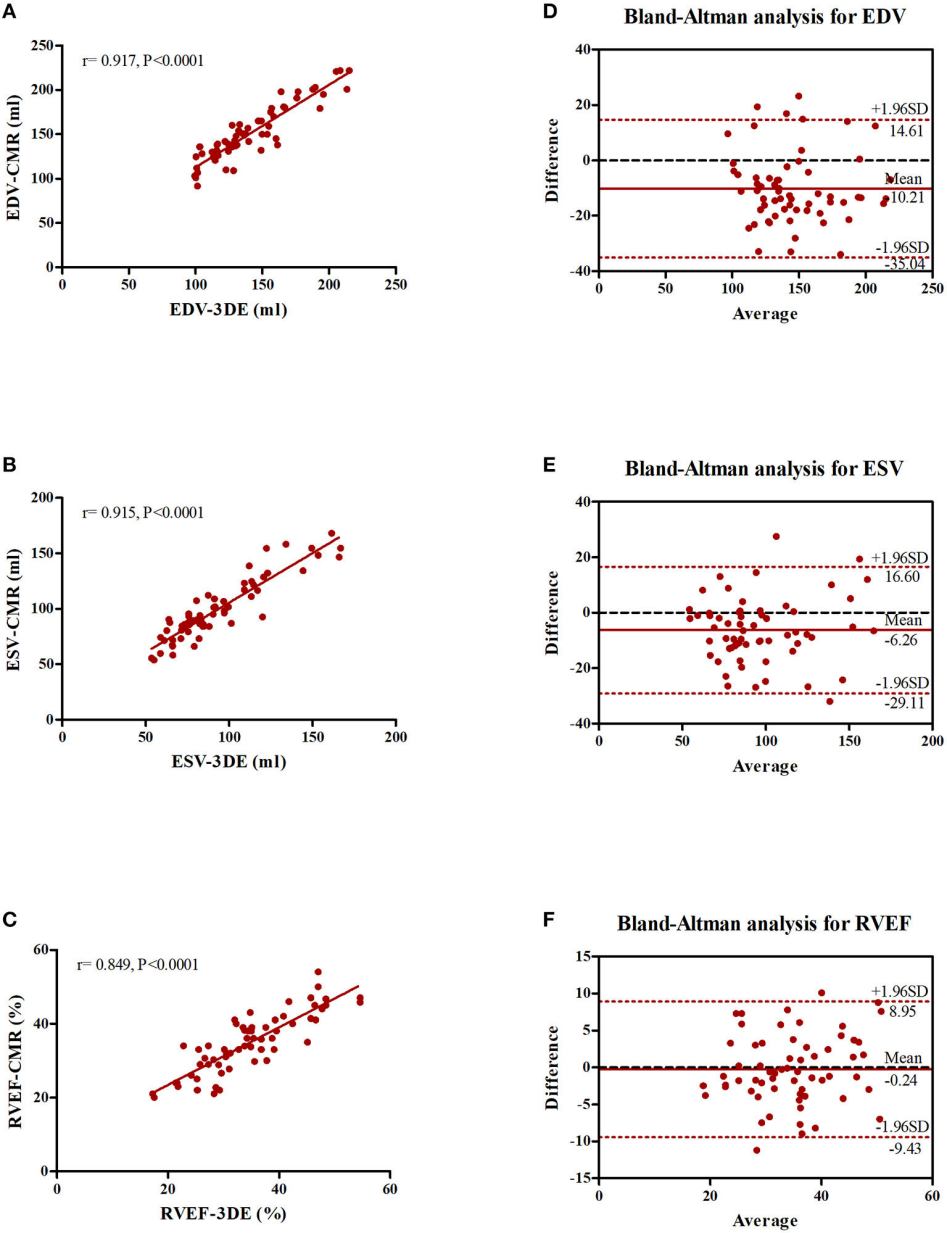
Correlation and Bland-Altman analysis of parameters measured by ML-based 3DE and CMR. **(A)** Correlation between RVEDV measured by ML-based 3DE and CMR. **(B)** Correlation between RVESV measured by ML-based 3DE and CMR. **(C)** Correlation between RVEF measured by ML-based 3DE and CMR. **(D)** Bland-Altman analyses of RVEDV measured by ML-based 3DE and CMR. **(E)** Bland-Altman analysis of RVESV measured by ML-based 3DE and CMR. **(F)** Bland-Altman analysis of RVEF measured by ML-based 3DE and CMR.

In the subset of patients who underwent CMR, ML-based 3DE measurements and CMR reference values had an excellent correlation for all parameters, as reflected by *r*-values of 0.917 for RVEDV, 0.915 for RVESV, and 0.849 for RVEF (all *P* < 0.001). The 3DE measurements were appreciably accurate as reflected by biases of −10.21 ± 12.66 ml for RVEDV (95% CI, −35.04~14.61), −6.26 ± 11.66 ml for RVESV (95% CI, −29.11~16.60), and −0.24 ± 4.69% for RVEF (95% CI, −9.43~8.95), although, the ML-based software algorithm slightly underestimated all three parameters compared to CMR (Figure 4).

### Prognostic Value of RVEF

The mean follow-up time was 19.7 months (interquartile range, 0.5–54 months), and 72 patients (47.7%) experienced adverse clinical outcomes during this follow-up period: 66 patients were hospitalized because of disease aggravation, of which 45 were hospitalized due to decompensated right heart failure, 13 underwent balloon pulmonary angioplasty, and eight had pulmonary endarterectomy. In addition, six patients died of PH-related causes outside the hospital.

The indicators of RV function and adverse clinical outcomes in the 151 patients are shown in Table 2. Except for RVESV and longitudinal diameter, there were significant differences in RV function and structural parameters in patients with or without clinical adverse outcomes. Patients with adverse clinical outcomes had higher RA pressure, mean pulmonary artery pressure, and pulmonary vascular resistance index, but had a lower cardiac index.

Univariate Cox proportional hazards analysis revealed that 6MWD and cardiac index were predictors of adverse clinical outcomes, the latter of which included RVEF, RVLS (Free wall), TAPSE, and RV fractional area change. Multivariate Cox proportional hazards analysis showed that ML-based 3DE RVEF and 6MWD had strong value for predicting adverse clinical outcomes (Table 3). Kaplan-Meier survival curves were generated, showing the relationship between the quality of RVEF. We combined the **two** interquartile ranges in the middle of a group (highest quartile > 44.6%, middle quartile 30.3–44.6%, lowest quartile < 30.3%) and then analyzed the correlation with clinical deterioration. We found that a decrease in RVEF increased the risk of clinical deterioration, and that the prognosis of patients with an RVEF < 30.3% was poorer compared to patients with an RVEF > 44.6% (Supplementary Figure 1).

**Table 3:** Cox regression analysis of adverse clinical outcomes.

**Variables**	**Univariate analysis**		**Multivariate analysis**	
	**HR (95% CI)**	***P*** **value**	**HR (95% CI)**	***P*** **-value**
Age	1.002 (0.99–1.002)	0.766		
Sex^*^	1.049 (0.737–1.494)	0.789		
Body surface area	1.019 (0.978–1.062)	0.357		
WHO functional class^*^	0.788 (0.573–1.085)	0.144		
6-min walk distance	1.885 (1.346–2.64)	**0.0002**	1.536 (1.067–2.211)	**0.021**
Echocardiographic data				
RVEF	2.035 (1.414–2.929)	**0.0001**	1.576 (1.046–2.372)	**0.030**
RVLS (Free wall)	2.128 (1.489–3.04)	**0.0001**	1.552 (0.990–2.432)	0.055
TAPSE	1.611 (1.149–2.258)	**0.006**		
RV index of myocardial performance	1.393 (0.963–2.015)	0.078		
RV fractional area change	1.827 (1.249–2.673)	**0.002**	1.332(0.871–2.036)	0.186
S'	1.37 (0.989–1.897)	0.058		
Hemodynamics data				
Mean PA pressure	1.924 (0.268–13.824)	0.515		
Cardiac index	1.66 (1.172–2.352)	**0.004**	1.161 (0.783–1.722)	0.457
Pulmonary vascular resistance	1.16 (0.7–1.922)	0.565		

*HR, hazard ratio per unit increase in the parameter measured;*

* *HR per category in the measured parameter. Bold values indicate statistical significance of the tested parameters*.

**Figure 5:**
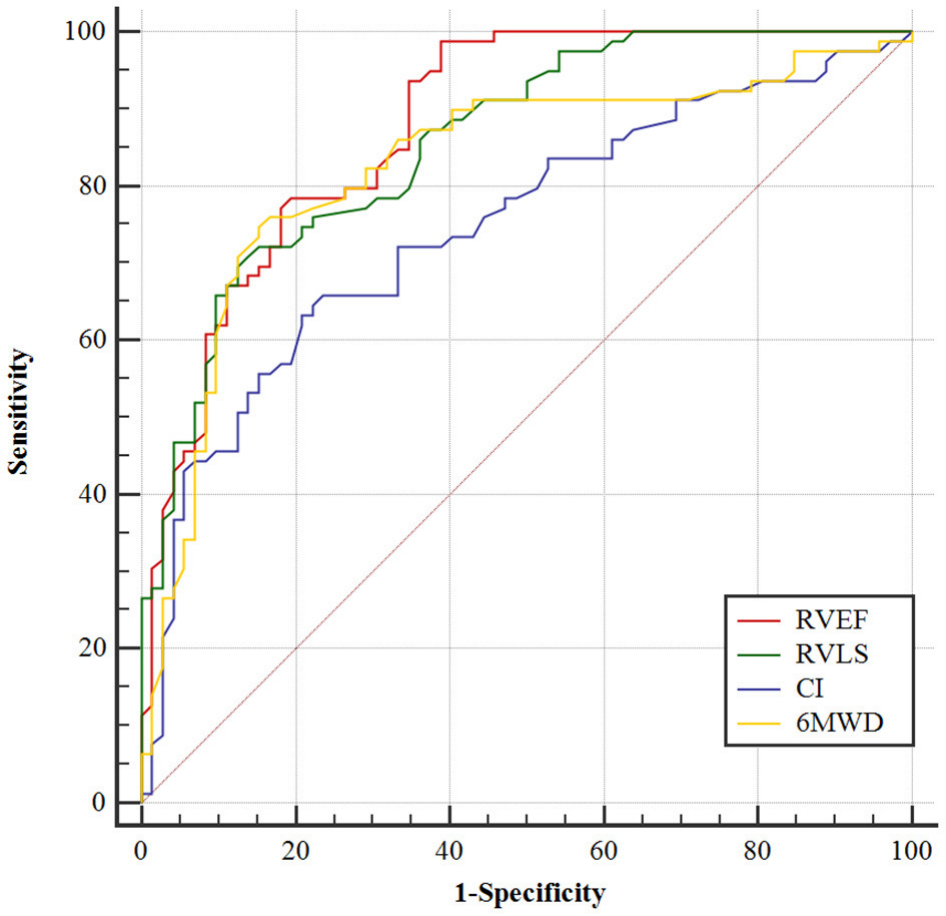
ROC analysis of RAFi. AUC, area under the curve. Receiver operating characteristic curve analysis demonstrating the ability of RVEF, RVLS, cardiac index, and 6MWD to predictive adverse clinical outcomes of CTEPH patients.

**Figure 6:**
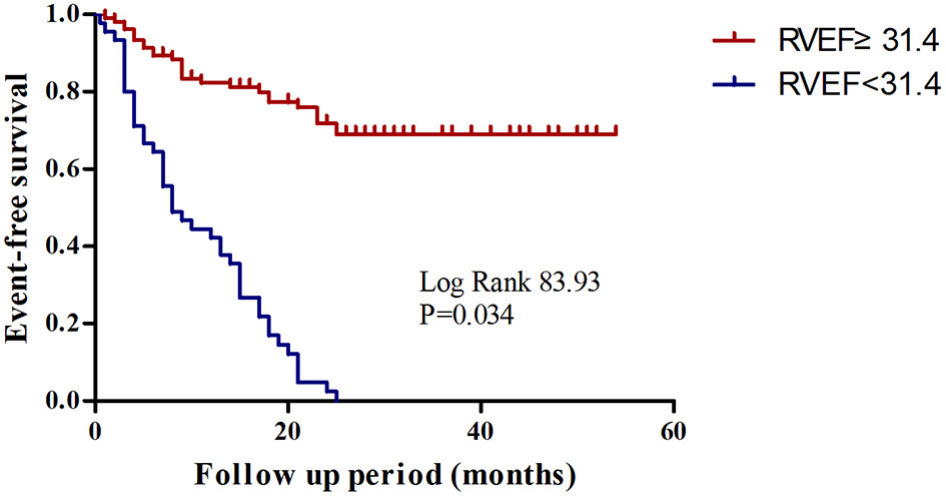
Kaplan-Meier survival analysis for CTEPH patients with a RVEF < 31.4 and ≥ 31.4%.

The best cutoff values for the most important parameters are shown in Table 4. ROC analysis revealed that an RVEF [area under the curve = 0.878; (95% CI, 0.825–0.932)] of 31.4% was the best cutoff value, with a sensitivity of 98.7% and a specificity of 61.1% (Figure 5). Patients with an RVEF < 31.4% had a 4.5-fold increased risk of clinical deterioration (log-rank *P* = 0.034, Figure 6). The addition of RVEF resulted in an increase in C statistic (from 0.639 to 0.78, *P* < 0.0001) (Table 5).

**Table 4:** Survival ROC analysis of the optimal cutoff values of tested parameters.

**Variables**	**Optimal cutoff**	**AUC**	**95% CI**	***P*** **-value**	**Sensitivity (%)**	**Specificity (%)**	**PPV (%)**	**NPV (%)**
RVEF (%)	31.4	0.878	0.825–0.932	<0.0001	98.7	61.1	97.8	73.6
RVLS (Free wall) (%)	−18.4	0.859	0.802–0.916	<0.0001	87.5	69.6	72.4	85.9
RV fractional area change (%)	29.6	0.810	0.74–0.879	<0.0001	69.6	86.1	72.1	84.6
6-min walk distance (m)	388.5	0.838	0.771–0.904	<0.0001	75.9	84.7	77.2	84.7
Cardiac index (L/min per m^2^)	2.6	0.747	0.668–0.825	<0.0001	63.3	79.2	66.3	76.9

*AUC, Area under the curve; NPV, negative predictive value; PPV, positive predictive value*.

**Table 5:** Additive value of RV EF (≥31.4%) predicts clinical outcomes.

**Variables**	**C index**	***Se***	***P*** **-value**
6-min walk distance	0.69	0.028	<0.0001
Cardiac index	0.639	0.026	<0.0001
RVEF	0.708	0.026	<0.0001
6-min walk distance + RVEF	0.767	0.027	<0.0001
Cardiac index + RVEF	0.747	0.027	<0.0001
6-min walk distance + cardiac index	0.726	0.028	<0.0001
6-min walk distance + cardiac index + RVEF	0.78	0.027	<0.0001

## Discussion

CTEPH patients are prone to have impaired RV function compared with normal subjects. In this prospective study, we introduced ML-based 3DE quantification of RVEF as a clinical means for predicting adverse clinical outcomes in CTEPH patients. We obtained the novel 3DE index, which easily combined information on RV volumes and RV function. We found that ML-based 3DE RVEF was a significant prognostic predictor for CTEPH patients.

Because of the shape of the RV and its physical position in the chest, traditional echocardiography for the evaluation of cardiac remodeling for CTEPH patients remains challenging. To remedy the limitation of conventional echocardiography, 2DE was developed to use multiple views from different transducer locations to quantify RV size and function. Previously, we demonstrated that RT3DE was a viable, non-invasive, and accurate method for assessing RV systolic function in PH patients (14). In this study, we demonstrated that ML-based 3DE RVEF had significant prognostic capability for CTEPH patients and had advantages over those established risk factors.

Measurement of RV size and function is important for the diagnosis and prognosis of CTEPH patients. Currently, CMR is considered the gold standard for assessing RV size and function. However, its limited availability and high cost do not permit routine evaluation and follow-up of these patients. Echocardiography is still valuable for the diagnosis and follow-up of CTEPH patients and can provide functional information that is not available with CMR. ML-based 3DE RVEF, as a simple parameter, may be more useful in routine clinical management of CTEPH patients.

In our CTEPH patient cohort, RVEF showed a correlation with basic clinical parameters, including cardiac index, 6MWD, and NT-proBNP. Our study demonstrated that CTEPH patients had a larger RV volume with a low RVEF and longitudinal strain compared with healthy controls. In addition, we found that RVEF, which was obtained by both echocardiography and CMR, correlated with RV functional parameters. More importantly, RVEF had a reinforced value in assessing disease severity because of the relation to cardiac index, 6MWD, and NT-proBNP. In line with our findings, a previous study reported that an increase in the RV/LV diameter ratio and high B-type natriuretic peptide levels were predictive of CTEPH development (15).

CTEPH is a rare but complex pathophysiological disease characterized by chronic thrombo-mechanical obstruction, RV dysfunction, and secondary pulmonary artery lesions (16). A recent study showed that the 1- and 5-year survival rates of patients with thromboembolic diseases and right heart failure were 71 and 49%, respectively (17). A 10-year prognostic study of CTEPH patients showed that worse final NYHA FC, inoperability, lower 6MWD at follow-up, higher NT-proBNP at follow-up, and reduced TAPSE are prognostic markers of poor outcomes of CTEPH patients (18). In the present study, we found that ML-based 3DE RVEF was a strong predictor of adverse clinical outcomes in CTEPH patients, with strong discriminative capacity as revealed by survival ROC analysis.

Cardiac remodeling is defined as changes in RV shape, size, and function of the heart. RV morphology is more complex than LV morphology. Previously, Addetia et al. (19) partitioned the trabecular section into body and apical components to assess changes in RV deformation at the level of the apex, as the apex appears to remodel distinctively on 2D imaging in pulmonary arterial hypertension (PAH) patients. In the present study, we used ML-based 3DE to assess RV function in CTEPH patients. We found that CTEPH patients with adverse clinical outcomes had greater enlargement of RV, lower RV function, and a higher incidence of pericardial effusion. It is worth noting that RV self-compensatory adaptation can maintain normal RV function for a short period of time, but this adaptation cannot be sustained long-term. Furthermore, progressive remodeling of the RV vascular bed results in its maladaptive remodeling. These pathophysiological changes are manifested as eccentric hypertrophy, RV expansion, decreased RV contractility, diastolic dysfunction, and myocardial fibrosis. RV failure due to the progressive dysfunction is the main cause of death for CTEPH patients (20). Our results showed that decreased RVEF increased the risk of clinical deterioration in CTEPH patients, and that patients with RVEF < 30.3% showed poor prognosis. Therefore, we should routinely measure RVEF in the clinic, pay close attention to decreases in right ventricular function in CTEPH patients, timely adjust the treatment plan when RVEF decreases, and consider the intervention of PEA or BPA as soon as possible when appropriate.

In the subset of our study cohort who underwent CMR, the ML-derived 3DE measurements significantly correlated with CMR reference values for all parameters. Despite association with CMR-derived RVEF, widely used parameters such as TAPSE and S' only assess the lateral tricuspid valve ring. 2D speckle tracking echocardiography has been used to quantitatively determine RV myocardial deformation, but mainly focuses on longitudinal strain. In this study, we demonstrated the feasibility and high value of 3DE analysis to estimate RVEF and volumes in healthy volunteers and CTEPH patients using technical software. Our study showed that RV strain patterns gradually worsened in CTEPH patients and thus held an independent prognostic value. In particular, ML-based 3DE RVEF predicted the adverse clinical outcomes independent of those established prognostic markers, suggesting that it reflected additional information on cardiac physiology that was not available for other functional or imaging parameters. Consistent with our findings, Jone et al. (21) showed the prognostic significance of 3D RV functional indices in pediatric PH patients, and that 3D RVEF was a significant outcome predictor for these patients.

In the present study, we also found that RVLS (Free wall) was an independent predictor of adverse clinical outcomes of CTEPH patients. RVLS indirectly but sensitively reflects RV function and hemodynamic changes in PH patients (22). Mortality in PH patients is affected by RV adverse remodeling, RV dilatation, and reduction in RVEF and/or AS. Moceri et al. (23) reported the independent prognostic role of RV AS. Meanwhile, RV segmental and global strain could help better stratify risk in PH patients. Traditional indicators of RV systolic function, including RVEF, may be maintained within the normal range despite the presence of PH with RV dysfunction. Thus, in PH patients, RV strain can detect resting RV dysfunction early in the course of the disease and may also serve as a marker of recessive RV systolic dysfunction (24). The ultimate challenge is understanding which balloon pulmonary angioplasty (BPA) has the best long-term outcomes for CTEPH patients (25). Patients with a low RVLS receive limited benefit from BPA treatment, and RVLS may be used as a valuable non-invasive parameter to predict the efficacy of BPA in CTEPH patients (26).

### Study Limitations

One major limitation of our study was the number of patients. As a result, this study included a relatively small number of adverse clinical outcomes, which prevented broader statistical analysis, especially for hard endpoints such as death. Also, due to the RV enlargement in CTEPH patients, the software automatic tracking system was not able to accurately identify the endocardial structure of the RV free wall if the RV expansion was severe or the display of the RV free wall was not clear. Nevertheless, we successfully captured quality images at the end of the exhalation period. In addition, because of the mentioned limitation, the RV volume measured by 3DE was smaller than that obtained by CMR. Finally, although, our study had a median follow-up time of 19.7 months, future studies should extend the follow-up time to ensure that the proportion of patients with poor prognosis due to right heart failure can be distinguished.

## Conclusion

ML-based 3DE RVEF is closely associated with RV systolic function and has a greater prognostic value than other recognized predictors for CTEPH patients. This new technique can help better stratify the risk of developing adverse clinical outcomes among CTEPH patients. Our study highlights the value of RVEF in risk assessment of CTEPH patients, but multi-center and larger cohort studies are needed to further corroborate our findings.

## Data Availability Statement

The raw data supporting the conclusions of this article will be made available by the authors, without undue reservation.

## Ethics Statement

The studies involving human participants were reviewed and approved by Ethics Committee of Beijing Chao-yang Hospital. The patients/participants provided their written informed consent to participate in this study.

## Author Contributions

YL and LL conceived and designed research. YL, YY, JG, XZ, and ZJ collected data and conducted research. LL and DZ analyzed and interpreted data. YL and DG wrote the initial paper. XL and YY revised the paper. YL had primary responsibility for final content. All authors read and approved the final manuscript.

## Conflict of Interest

The authors declare that the research was conducted in the absence of any commercial or financial relationships that could be construed as a potential conflict of interest.

## Publisher's Note

All claims expressed in this article are solely those of the authors and do not necessarily represent those of their affiliated organizations, or those of the publisher, the editors and the reviewers. Any product that may be evaluated in this article, or claim that may be made by its manufacturer, is not guaranteed or endorsed by the publisher.

## Supplementary Material

Supplementary Figure 1 Impact of RVEF on clinical failure of CTEPH patients. Kaplan-Meier survival analysis according to quartiles of RVEF, after combining the two middle quartiles, finally generating three groups.

## Abbreviations

AUC: area under the curve
BSA: body surface area
CI: Cardiac index
CMR: cardiac magnetic resonance
CT: computed tomography
CTEPH: chronic thromboembolic pulmonary hypertension
EI: eccentricity index
ICCs: intraclass correlation coefficients
LVD: left ventricular diameter
LVEF: left ventricular ejection fraction
ML: machine learning
MRI: magnetic resonance imaging
NT-proBNP: N-terminal pro-brain natriuretic peptide
PAP: pulmonary artery pressure
PAWP: pulmonary wedge pressure
PVR: pulmonary vascular resistance
RAP: right arterial pressure
ROC: receiver operating characteristic
RV: right ventricular
RV EDA: RV end-diastolic area
RVEF: right ventricular ejection fraction
RV ESA: RV end-systolic area
RVFAC: RV fractional area change
RIMP: RV index of myocardial performance
3DE: three-dimensional echocardiography
2D-STE: two-dimensional speckle tracking strain echocardiography
TAPSE: tricuspid annular plane systolic excursion
WHO: World Health Organization
6MWT: 6-minute walk test.

## References

[1] Wilkens H Konstantinides S Lang IM Bunck AC Gerges M Gerhardt F. Chronic thromboembolic pulmonary hypertension (CTEPH): updated recommendations from the Cologne Consensus Conference 2018. Int J Cardiol. (2018) 272S:69–78. 10.1016/j.ijcard.2018.08.079 30195840

[2] Pepke-Zaba J Delcroix M Lang I Mayer E Jansa P Ambroz D. Chronic thromboembolic pulmonary hypertension (CTEPH): results from an international prospective registry. Circulation. (2011) 124:1973–81. 10.1161/CIRCULATIONAHA.110.015008 21969018

[3] Puengpapat S Pirompanich P. Incidence of chronic thromboembolic pulmonary hypertension in Thammasat University Hospital. Lung India. (2018) 35:373–8. 10.4103/lungindia.lungindia_158_18 30168454 PMC6120324

[4] Tapson VF Humbert M. Incidence and prevalence of chronic thromboembolic pulmonary hypertension: from acute to chronic pulmonary embolism. Proc Am Thorac Soc. (2006) 3:564–7. 10.1513/pats.200605-112LR 16963534

[5] Ende-Verhaar YM Cannegieter SC Vonk Noordegraaf A Delcroix M Pruszczyk P Mairuhu AT. Incidence of chronic thromboembolic pulmonary hypertension after acute pulmonary embolism: a contemporary view of the published literature. Eur Respir J. (2017) 49:1601792. 10.1183/13993003.01792-2016 28232411

[6] Simpson CE Hassoun PM. Myocardial fibrosis as a potential maladaptive feature of right ventricle remodeling in pulmonary hypertension. Am J Respir Crit Care Med. (2019) 200:662–3. 10.1164/rccm.201906-1154ED 31216171 PMC6775878

[7] Muraru D Spadotto V Cecchetto A Romeo G Aruta P Ermacora D. New speckle-tracking algorithm for right ventricular volume analysis from three-dimensional echocardiographic data sets: validation with cardiac magnetic resonance and comparison with the previous analysis tool. Eur Heart J Cardiovasc Imaging. (2016) 17:1279–89. 10.1093/ehjci/jev309 26647080

[8] Laser KT Karabiyik A Körperich H Horst JP Barth P Kececioglu D. Validation and reference values for three-dimensional echocardiographic right ventricular volumetry in children: a multicenter study. J Am Soc Echocardiogr. (2018) 31:1050–63. 10.1016/j.echo.2018.03.010 29908725

[9] Medvedofsky D Addetia K Patel AR Sedlmeier A Baumann R Mor-Avi V. Novel approach to three-dimensional echocardiographic quantification of right ventricular volumes and function from focused views. J Am Soc Echocardiogr. (2015) 28:1222–31. 10.1016/j.echo.2015.06.013 26237996

[10] Genovese D Rashedi N Weinert L Narang A Addetia K Patel AR. Machine learning-based three-dimensional echocardiographic quantification of right ventricular size and function: validation against cardiac magnetic resonance. J Am Soc Echocardiogr. (2019) 32:969–77. 10.1016/j.echo.2019.04.001 31174940

[11] Stoel B. Use of artificial intelligence in imaging in rheumatology - current status and future perspectives. RMD Open. (2020) 6. 10.1136/rmdopen-2019-001063 31958283 PMC6999690

[12] Galiè N Humbert M Vachiery JL Gibbs S Lang I Torbicki A. 2015 ESC/ERS guidelines for the diagnosis and treatment of pulmonary hypertension: the joint task force for the diagnosis and treatment of pulmonary hypertension of the European Society of Cardiology (ESC) and the European Respiratory Society (ERS): endorsed by: Association for European Paediatric and Congenital Cardiology (AEPC), International Society for Heart and Lung Transplantation (ISHLT). Eur Heart J. (2016) 37:67–119. 10.1093/eurheartj/ehv317 26320113

[13] Lang RM Badano LP Mor-Avi V Afilalo J Armstrong A Ernande L. Recommendations for cardiac chamber quantification by echocardiography in adults: an update from the American Society of Echocardiography and the European Association of Cardiovascular Imaging. Eur Heart J Cardiovasc Imaging. (2015) 16:233–70. 10.1093/ehjci/jev014 25712077

[14] Li Y Wang Y Zhai Z Guo X Yang Y Lu X. Real-time three-dimensional echocardiography to assess right ventricle function in patients with pulmonary hypertension. PLoS ONE. (2015) 10:e0129557. 10.1371/journal.pone.0129557 26075788 PMC4468177

[15] Medrek S Safdar Z. Epidemiology and pathophysiology of chronic thromboembolic pulmonary hypertension: risk factors and mechanisms. Methodist Debakey Cardiovasc J. (2016) 12:195–8. 10.14797/mdcj-12-4-195 28289493 PMC5344468

[16] Ranka S Mohananey D Agarwal N Verma BR Villablanca P Mewhort HE. Chronic thromboembolic pulmonary hypertension-management strategies and outcomes. J Cardiothorac Vasc Anesth. (2020) 34:2513–23. 10.1053/j.jvca.2019.11.019 31883688

[17] Padang R Chandrashekar N Indrabhinduwat M Scott CG Luis SA Chandrasekaran K. Aetiology and outcomes of severe right ventricular dysfunction. Eur Heart J. (2020) 41:1273–82. 10.1093/eurheartj/ehaa037 32047900

[18] Küçükoglu MS Sinan Ü Y Yildizeli B. Ten-year outcome of chronic thromboembolic pulmonary hypertension patients in a tertiary center. Anatol J Cardiol. (2020) 23:105–9. 10.14744/AnatolJCardiol.2019.90329 32011330 PMC7040873

[19] Addetia K Maffessanti F Yamat M Weinert L Narang A Freed BH. Three-dimensional echocardiography-based analysis of right ventricular shape in pulmonary arterial hypertension. Eur Heart J Cardiovasc Imaging. (2016) 17:564–75. 10.1093/ehjci/jev171 26160404

[20] Simonneau G Torbicki A Dorfmüller P Kim N. The pathophysiology of chronic thromboembolic pulmonary hypertension. Eur Respir Rev. (2017) 26. 10.1183/16000617.0112-2016 28356405 PMC9488693

[21] Jone PN Schäfer M Pan Z Bremen C Ivy DD. 3D echocardiographic evaluation of right ventricular function and strain: a prognostic study in paediatric pulmonary hypertension. Eur Heart J Cardiovasc Imaging. (2018) 19:1026–33. 10.1093/ehjci/jex205 28950335

[22] Li Y Wang Y Meng X Zhu W Lu X. Assessment of right ventricular longitudinal strain by 2D speckle tracking imaging compared with RV function and hemodynamics in pulmonary hypertension. Int J Cardiovasc Imaging. (2017) 33:1737–48. 10.1007/s10554-017-1182-3 28553693

[23] Moceri P Duchateau N Baudouy D Schouver ED Leroy S Squara F. Three-dimensional right-ventricular regional deformation and survival in pulmonary hypertension. Eur Heart J Cardiovasc Imaging. (2018) 19:450–8. 10.1093/ehjci/jex163 28637308

[24] Ferrara F Zhou X Gargani L Wierzbowska-Drabik K Vriz O Fadel BM. Echocardiography in pulmonary arterial hypertension. Curr Cardiol Rep. (2019) 21:22. 10.1007/s11886-019-1109-9 30828743

[25] Auger WR. Surgical and percutaneous interventions for chronic thromboembolic pulmonary hypertension. Cardiol Clin. (2020) 38:257–68. 10.1016/j.ccl.2020.01.003 32284102

[26] Zhang X Guo D Wang J Gong J Wu X Jiang Z. Speckle tracking for predicting outcomes of balloon pulmonary angioplasty in patients with chronic thromboembolic pulmonary hypertension. Echocardiography. (2020) 37:841–9. 10.1111/echo.14709 32447819

